# Intestinal mucosal mitochondrial oxidative phosphorylation worsens with cirrhosis progression and is ameliorated with fecal microbiota transplantation

**DOI:** 10.1172/jci.insight.186649

**Published:** 2025-01-07

**Authors:** Jing Zeng, Derrick Zhao, Grayson Way, Andrew Fagan, Michael Fuchs, Puneet Puri, Brian C. Davis, Xuan Wang, Emily C. Gurley, Phillip B. Hylemon, Jian-Gao Fan, Masoumeh Sikaroodi, Patrick M. Gillevet, Huiping Zhou, Jasmohan S. Bajaj

**Affiliations:** 1 Virginia Commonwealth University and Richmond VA Medical Center, Richmond, Virginia, USA.; 2 Department of Gastroenterology, Xinhua Hospital, Shanghai Jiao Tong University School of Medicine, Shanghai, China.; 3 Stravitz-Sanyal Institute for Liver Disease & Metabolic Health, School of Medicine, Virginia Commonwealth University, Richmond, Virginia, USA.; 4 Microbiome Analysis Center, George Mason University, Manassas, Virginia, USA.

**Keywords:** Hepatology, Microbiology, Hepatitis, Innate immunity, Liver cancer

## Abstract

Cirrhosis, the end-stage of liver disease, progresses through altered gut-liver axis and microbial change. Here we show that intestinal mucosal mitochondrial oxidative phosphorylation, which affects intestinal barrier worsens with cirrhosis progression. This is ameliorated with fecal microbiota transplantation.

To the Editor: Cirrhosis is a critical global health concern because of its high mortality and morbidity rates and because of high incidence of obesity, alcohol consumption, and viral hepatitis (1, 2). This advanced liver disease disrupts the gut/liver axis, affecting normal physiological processes, including mitochondrial oxidative phosphorylation (OXPHOS) activity in the intestinal mucosa, which is crucial for cellular energy production. Dysfunctional OXPHOS can lead to exacerbated tissue hypoxia and energy deficits. Additionally, alterations in the gut microbiota influence metabolic and immune responses, significantly affecting liver disease outcomes (3). Furthermore, the gut/liver axis, which encompasses the bidirectional relationship between the intestinal microbiota and liver health, plays a pivotal role in the progression of cirrhosis. Alterations in gut microbiota composition have been linked to variations in metabolic and immune responses that significantly affect liver disease outcomes. In this context, fecal microbiota transplantation (FMT) emerges as a therapeutic approach aimed at restoring a healthy microbiota balance, potentially modulating mitochondrial function, and ameliorating cirrhosis progression (4). It is important to understand the intricate relationship among intestinal mucosal OXPHOS, changes in gut microbiota, and the impact of FMT in cirrhosis.

We conducted a cross-sectional study involving 32 age-balanced men divided into 3 groups: healthy controls, patients with compensated cirrhosis, and patients with decompensated cirrhosis. Participants underwent endoscopic procedures to obtain biopsies from the duodenum (DUOD) and ascending (ASCEND) colon. These samples were analyzed for mitochondrial OXPHOS gene expression using quantitative PCR and NanoString (Bruker) technologies. Additionally, a subset of patients with decompensated cirrhosis previously enrolled in a randomized clinical trial received FMT capsules from a single donor (5). Changes in OXPHOS gene expression were measured before and after FMT in the DUOD to evaluate the impact of microbiota modulation on mitochondrial function. The study also included comprehensive bioinformatics analysis to assess correlations between changes in gut microbiota composition, clinical parameters such as the Model for End-Stage Liver Disease scores, and mitochondrial activity. Further details on methods, including patient details, are in Supplemental Methods, Supplemental Table 1, and Supplemental Figures 1 and 2; supplemental material available online with this article; https://doi.org/10.1172/jci.insight.186649DS1 Sex as a biological variable was considered, but we ended up including only 1 woman in the entire study because of the population at the Veterans Affairs hospital. In the DUOD of patients with cirrhosis compared with healthy controls, Kyoto Encyclopedia of Genes and Genomes (KEGG) pathway analysis of differentially expressed genes (DEGs) indicated involvement in OXPHOS (Figure 1A). In patients with decompensated cirrhosis, there were more pronounced alterations (Figure 1B). Additionally, the study revealed significant upregulation of inflammation-related genes and nuclear-encoded OXPHOS genes in patients with compensated and decompensated cirrhosis compared with healthy controls (|fold-change| > 1.5; *P* < 0.05) (Figure 1, C and D), correlating strongly with the gut microbiota composition changes and the severity of cirrhosis (*P* < 0.05) (Supplemental Figure 3). This upregulation was particularly pronounced in the decompensated cirrhosis group, indicating severe inflammation and mitochondrial dysfunction. Following FMT, there was a significant reduction in OXPHOS gene expression in the DUOD of patients (*P* < 0.05), suggesting an improvement in mitochondrial function and a potential restoration of the intestinal barrier. Additionally, patients who received FMT showed enrichment in pathways associated with inhibited reactive oxygen species production and OXPHOS, which were higher at baseline (*P* < 0.05, Figure 1, E and F).

This study investigates the correlation between intestinal mucosal OXPHOS and inflammation in patients with cirrhosis, their relationship to microbiota and the progression of liver disease, as well as modulation after successful FMT. The analysis reveals changes in genes related to OXPHOS and inflammation, which are particularly pronounced in patients with decompensated cirrhosis. Moreover, the study underscores the importance of gut microbiota composition in the development of cirrhosis, with certain bacterial genera showing correlations with the expression of genes related to OXPHOS and inflammation. We hypothesized that during the development and progression of cirrhosis, intestinal inflammation, altered blood flow, and resulting tissue hypoxia affect luminal oxygen availability (6), potentially disadvantaging obligately anaerobic bacteria, such as *Megasphaera* and *Fusicatenibacter*. In contrast, such conditions could favor facultative anaerobes, such as *Pasteurella*. The findings regarding changes in OXPHOS genes expression and microbiota in both the small and large intestines offer valuable insights into the potential mechanisms contributing to the progression of cirrhosis. FMT using oral capsules restored small intestinal mitochondrial OXPHOS activity in the duodenal mucosa, highlighting the potential interaction between microbial change and intestinal inflammation in cirrhosis.

Future research should seek to replicate these findings in larger and more diverse cohorts to verify the consistency of the results and assess the long-term effects of FMT on mitochondrial function and clinical outcomes in cirrhosis. Additionally, mechanistic studies are crucial for elucidating the specific pathways through which alterations in the microbiota influence mitochondrial dynamics. Identifying microbial species that affect these processes will further our understanding of their roles in liver disease progression and the potential for targeted microbiota-based therapies. In conclusion, this study has explored the associations among intestinal mucosal OXPHOS, inflammation, and dysbiosis in patients with cirrhosis, shedding light on their interconnected roles in the progression of liver disease and underscoring the therapeutic possibilities of microbiota-based interventions. These findings highlight a complex interplay between intestinal mucosal OXPHOS function, cirrhosis, and FMT. However, it is important to recognize that these results are primarily correlational. A deeper understanding of the gut/microbiota/liver nexus and its impact on cellular energy pathways could pave the way for future research to develop therapeutic strategies that go beyond symptom management and potentially modify the disease trajectory in cirrhosis. The insights gained from this study broaden our understanding of cirrhosis pathogenesis and underscore the importance of exploring innovative treatment approaches.

## Supplementary Material

Supplemental data

## Figures and Tables

**Figure 1:**
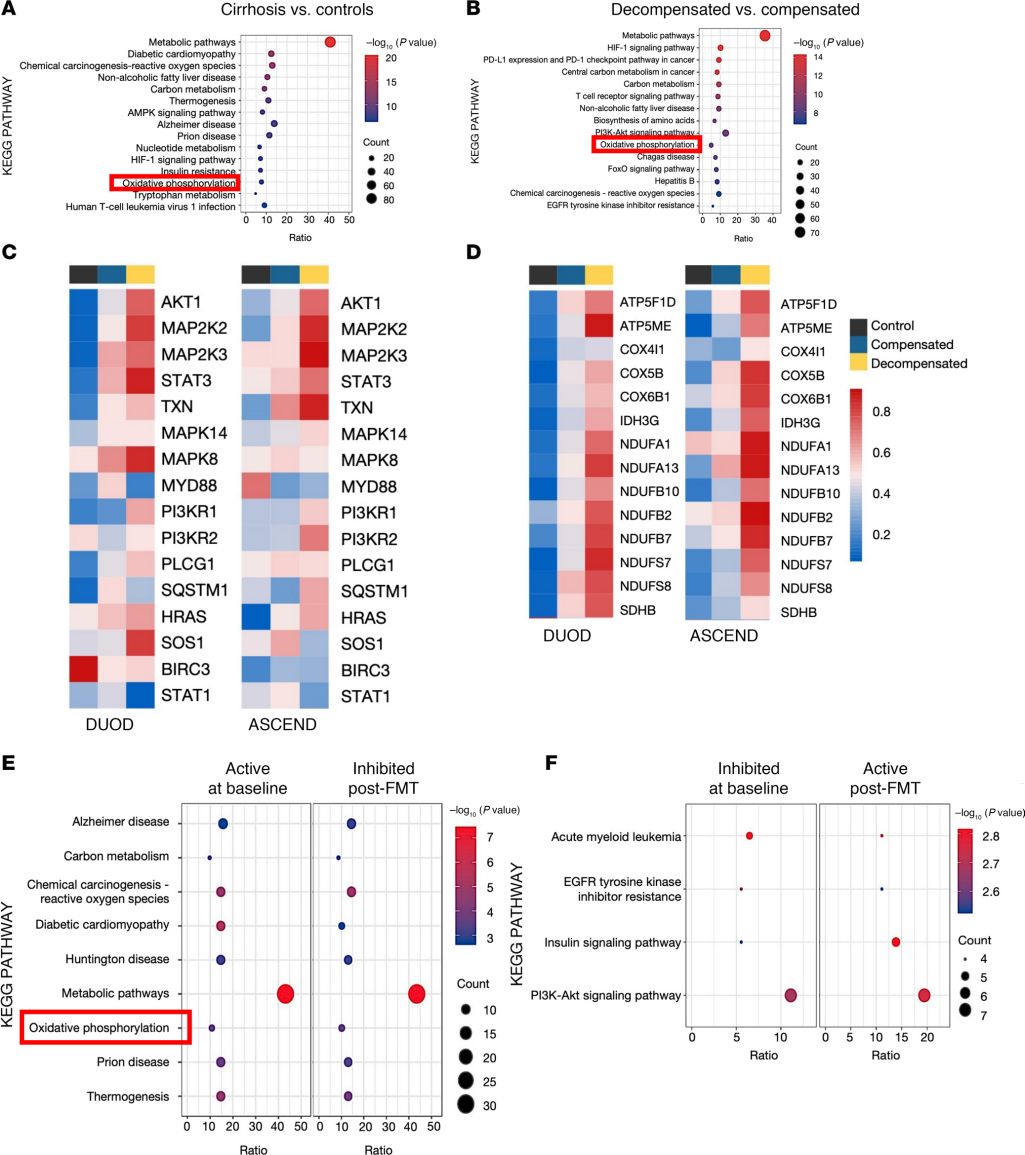
Intestinal mucosal changes in cirrhosis using cross-sectional and post-FMT approaches. (**A** and **B**) Functional gene analysis in the DUOD: KEGG pathway analyses differentiate between healthy controls and patients with cirrhosis (**A**), as well as between compensated and decompensated stages of cirrhosis (**B**). (**C** and **D**) Gene expression profiles in cirrhosis: Hierarchical clustering heatmaps display changes in inflammation-related (**C**) and OXPHOS-related gene expression (**D**) in the DUOD and ASCEND colon of patients across different stages of cirrhosis compared with healthy controls, utilizing NanoString nCounter metabolic, inflammation, and fibrosis mRNA panels. (**E** and **F**) KEGG pathway analysis of DEGs in post-FMT DUOD versus pre-FMT (baseline). (**E**) DEGs suppressed after FMT. (**F**) DEGs activated after FMT.

## Version 1 01/07/2025

In-Press Preview

## Version 2 02/24/2025

Electronic publication
