# Supplementary material for: Intestinal mucosal mitochondrial oxidative phosphorylation worsens with cirrhosis progression and is ameliorated with fecal microbiota transplantation
Source: JCI Insight. 2025 Jan 7;10(4):e186649. doi: 10.1172/jci.insight.186649 (PMC11949008; doi:10.1172/jci.insight.186649)
Supplement: Supplemental data [file jciinsight-10-186649-s002.pdf]

Intestinal Mucosal Mitochondrial Oxidative Phosphorylation worsens with Cirrhosis Progression and is ameliorated with FMT

Jing Zeng[1, 2], Derrick Zhao[1], Grayson Way[1], Andrew Fagan[3], Michael Fuchs[3], Puneet Puri[3], Brian C Davis[3], Xuan Wang[1], Emily C Gurley[1], Phillip B. Hylemon[1, 4], Jian-Gao Fan[2], Masoumeh Sikaroodi[5], Patrick M Gillevet[5], Huiping Zhou[1, 4], and Jasmohan S Bajaj[3, 4]

Supplement:

Supplemental Methods
Supplemental Table 1, Figures S1-S3

Supplemental Methods

Subject Recruitment

Thirty-two age-matched male participants were enrolled in the cross-sectional study. The subjects were categorized into three groups: healthy controls, compensated cirrhosis, or decompensated cirrhosis. Cirrhosis was diagnosed using one or more of the following methods: liver biopsy, evidence of varices, radiological signs in patients with chronic liver disease, clear signs of decompensation, or high transient elastography values (>12.5 Kpa). None of the patients were on absorbable antibiotics and only rifaximin use was allowed. Patients with decompensated cirrhosis experienced either ascites or hepatic encephalopathy, which was controlled with medication at the time of the study. Healthy controls were free from chronic diseases and were not on any prescription medications. Demographics, details of cirrhosis, and prior complications were recorded.

For the FMT study, a subset of the decompensated cirrhosis group was selected based on their participation in a prior randomized controlled trial (RCT) assessing the efficacy of oral capsular FMT. More details can be found in the prior publication but in summary, patients decompensated cirrhosis on lactulose and rifaximin were randomized 1:1 into receiving one-time capsules (n=10) at baseline and followed for 6 months with changes in microbiome composition and function(20) from the same donor who were enriched in *Lachnospiraceae* and *Ruminococcaceae*. All subjects underwent an esophagogastroduodenoscopy (EGD) at baseline and those who were randomized to active FMT underwent a repeat EGD at 15 days post-FMT. The study adhered to rigorous ethical guidelines, obtaining informed consent from all participants prior to their inclusion. Ethical approval for the study protocol was secured from the appropriate institutional review board (IRB) or ethics committee.

Endoscopic Procedures

Each participant in the cross-sectional study group underwent two simultaneous endoscopic procedures: (EGD) and colonoscopy. During these procedures, pinch biopsies were obtained from two distinct anatomical sites: the duodenum (DUOD) and the ascending colon (ASCEND). These sites were selected to examine differential gene expression patterns along the gastrointestinal tract. Rigorous procedures involving single-use biopsy forceps were used to prevent cross-contamination.

Participants in the FMT study group only underwent an EGD before the administration of FMT capsules and a repeat EGD 15 days after the initial procedure. Colonoscopies were not performed as part of this trial therefore ascending colon samples were not obtained.

Isolation of RNA and RNA Quality Control

Total RNA was isolated from the intestinal tissues of these subjects using TRIzol reagent (Thermo Fisher Scientific, Inc, Waltham, MA, USA) following the instructions. The purity and concentration of total RNA from each sample were measured using a NanoDrop ND-1000 instrument by calculating the OD260 value. The quality of the RNA was evaluated by electrophoresis on a denaturing agarose gel.

Identification of Differentially Expressed Genes

The gene profiles from samples of healthy controls, individuals with compensated cirrhosis, and those with decompensated cirrhosis, as well as before and after FMT, were measured using the NanoString nCounter® system. The data were analyzed using ROSALIND® (https://rosalind.bio/), which features a HyperScale architecture developed by ROSALIND, Inc. (San Diego, CA). Differentially expressed genes (DEGs) were identified based on statistical significance between two groups using a cutoff criterion of $P < 0.05$ and $|\text{fold change (FC)}| > 1.5$.

Functional Enrichment Analyses

Gene Ontology (GO) function and Kyoto Encyclopedia of Genes and Genomes (KEGG) pathway enrichment analyses were conducted on the identified DEGs. These analyses provided insights into the biological functions and pathways that were most affected by the observed gene expression changes. This step was essential for elucidating the molecular mechanisms underlying cirrhosis progression and its associated pathophysiological alterations.

Microbiome 16S rRNA Gene Sequencing

16S rRNA ribosomal RNA (rRNA) gene sequencing was performed from all mucosal pinch biopsy samples using published techniques. Individual microbiota at the family and genus levels were compared between controls and cirrhosis and within the cirrhosis group using Linear discriminant function effect size analysis (LEfSe). Correlation heatmaps were created related to specific genes of interest and microbiota in the mucosa using $p<0.05$ and $r>0.5$ or <-0.5 cut-off. **Data availability statement**

Data are available at GEO accession GSE285291. Access is at https://www.ncbi.nlm.nih.gov/geo/query/acc.cgi?acc=GSE285291 with token ejkvkqiynjqljkl

AUTHOR CONTRIBUTIONS

Jing Zeng: Data collection, data analysis, manuscript writing and editing. Derrick Zhao, Grayson Way, Emily C Gurley: Data collection, data analysis. Andrew Fagan, Michael Fuchs, Puneet Puri, Brian C Davis: Clinical data collection, patient management. Xuan Wang: Data analysis, statistical support. Phillip B. Hylemon: Manuscript writing, editing, and review. Jian-Gao Fan: Study concept, manuscript review and editing. Patrick Gillevet and Masoumeh Sikaroodi: Data collection, data analysis, manuscript writing and editing Huiping Zhou: Study concept and design, funding acquisition, study supervision, manuscript writing and editing. Jasmohan S Bajaj: Study concept and design, study conduct, funding acquisition, study supervision, data analysis, manuscript writing, editing and guarantor of the article.

ACKNOWLEDGEMENTS

Not applicable.

FUNDING

This study was supported by VA Merit I0CX001076, VA Merit I01CX002472, Richmond Institute for Veterans Research, VA Merit Award I01BX005730; Research Career Scientist Award IK6BX004477); VA ShEEP grants (1IS1BX005517-01 and 1 IS1 BX004777-01) National Institutes of Health Grant R01DK139587 and 2R56DK115377-05A1, 1R01DK104893, and 1P01CA275740.

Supplemental Table 1: Comparison of subjects in the study

	Cross-sectional*				FMT trial (only pre/post EGD)**		
	Control (n=4)	Compensated (n=4)	Decompensated (n=24)	P value	Pre (n=9)	Post (n=9)	P value
Age	57.3±6.3	59.8±7.6	63.1±5.2	0.15	63.3±4.2	-	-
Gender (men/women)	4/0	4/0	23/1	1.0	8/1	-	-
MELD	-	6.3±0.5	9.9±3.6	<0.001	9.4±2.7	8.9±3.1	0.72
PPI use	0	2 (50%)	21 (88%)	0.13	9 (100%)	9 (100%)	1.0
Albumin	-	3.7±0.2	3.3±0.5	0.01	3.3±0.8	3.5±0.7	0.58
Etiology(HCV, alcohol,MASH, others)	-	2/2/0/0	6/10/6/2	0.87	2/4/2/1	-	
Lactulose	-	0	22 (92%)	-	9 (100%)	9 (100%)	1.0
Rifaximin	-	0	24 (100%)	-	9 (100%)	9 (100%)	1.0
Ascites	-	0	18 (75%)	-	9 (100%)	9 (100%)	1.0

*: includes the baseline visit for all patients included in the FMT trial (placebo and FMT-assigned), **: Only the 9 patients in those assigned to FMT who has EGD pre and post are included. MASH: metabolic dysfunction-associated steatotic liver disease, HCV: hepatitis C Virus, MELD: model for end-stage liver disease, PPI: proton pump inhibitors, EGD: esophago-gastroduodenoscopy.

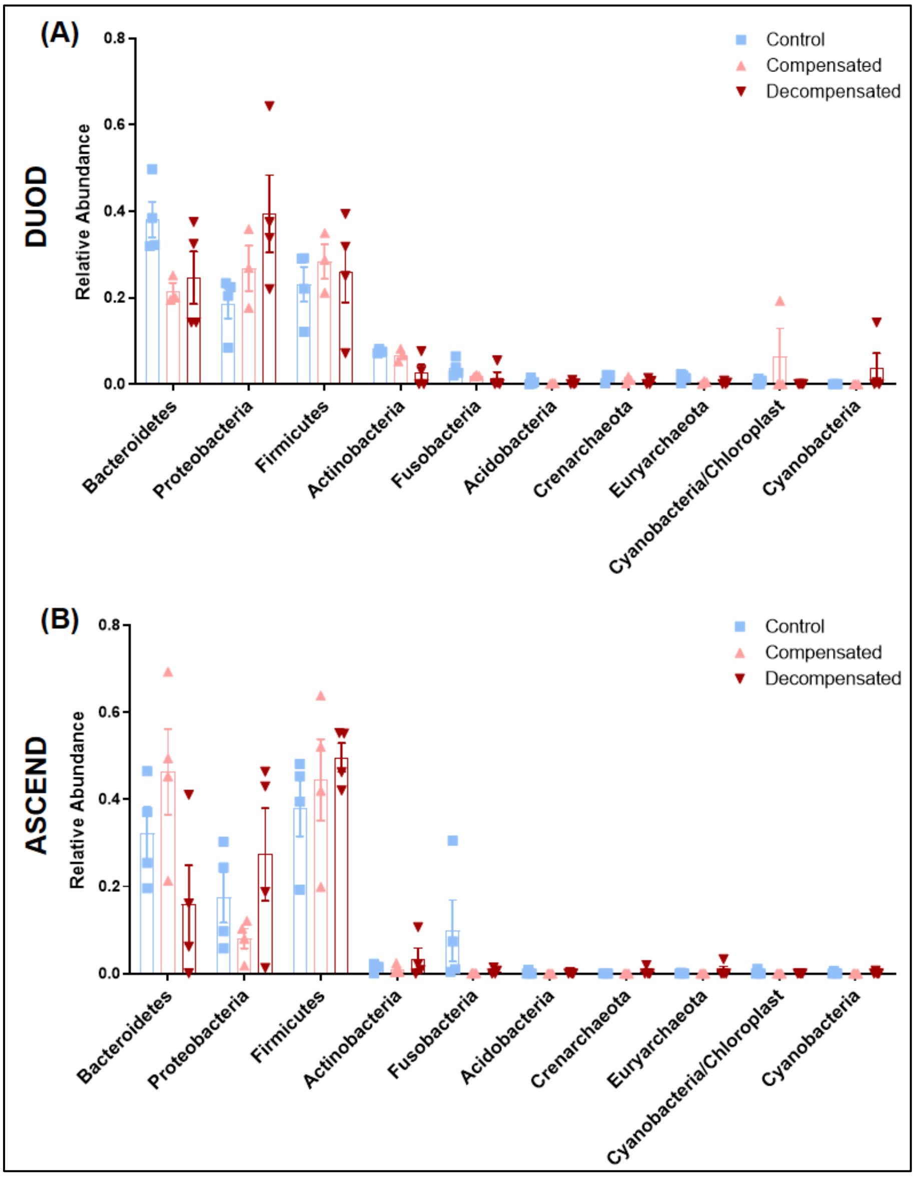

Supplemental Figure 1. The microbiome composition at phylum level in subjects with cirrhosis. (A) DUOD. (B) ASCEND. DUOD, duodenum; ASCEND, ascending colon.

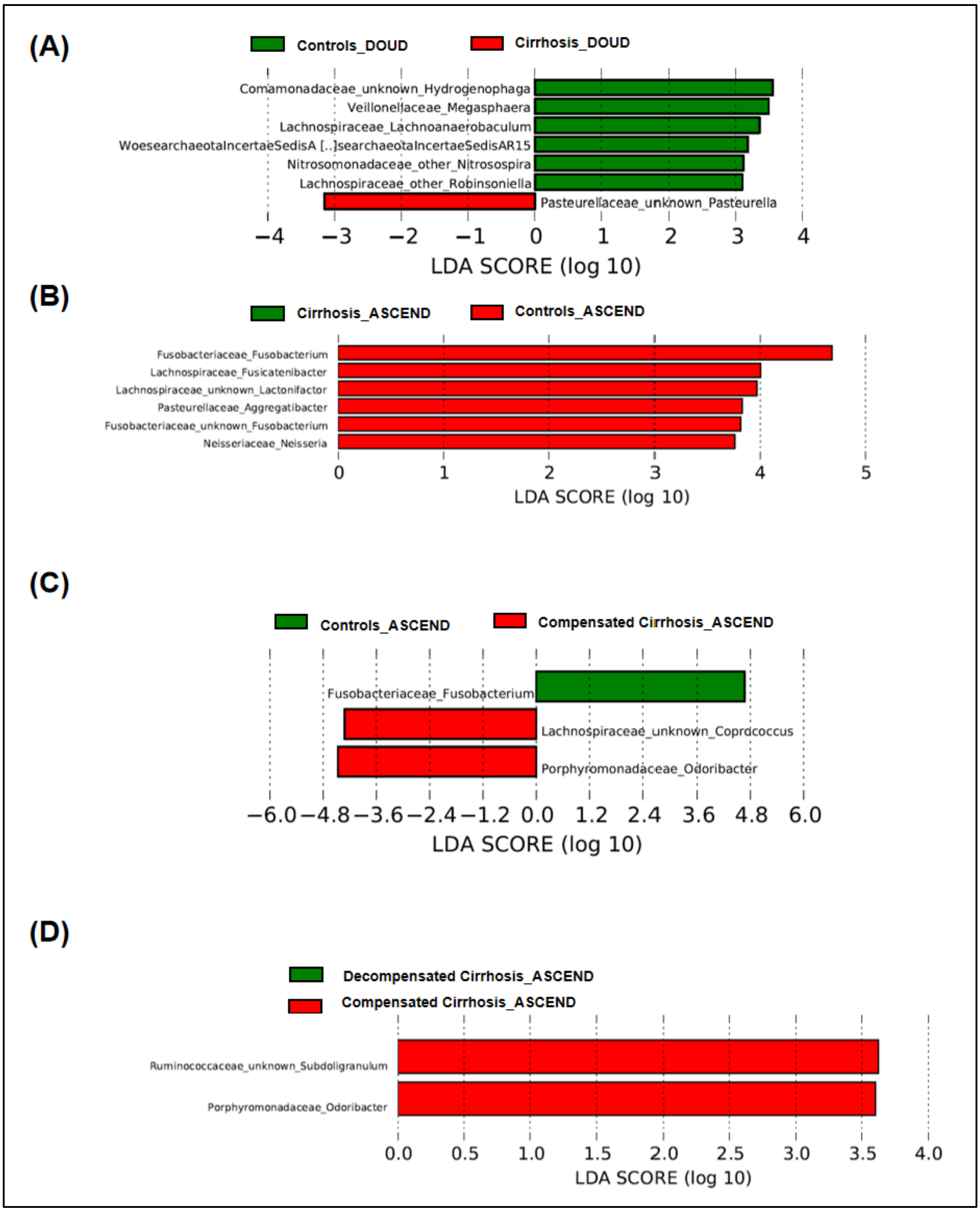

Supplemental Figure 2. Differences in the microbiota in the genera level.

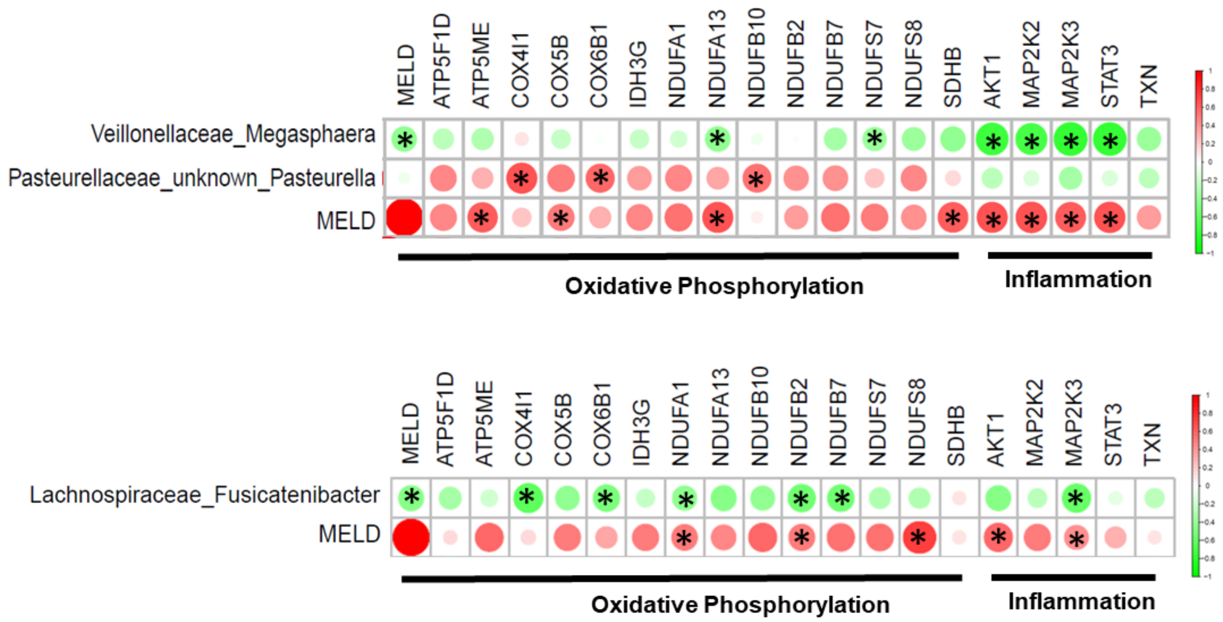

Supplemental Figure 3. Genera-level Microbiota and Gene Correlations:

Heatmap illustrating Spearman correlations between significant microbial genera and related gene changes and MELD scores in cirrhosis, *p<0.05.
